# Identification of two novel and one rare mutation in *DYRK1A* and prenatal diagnoses in three Chinese families with intellectual Disability-7

**DOI:** 10.3389/fgene.2023.1290949

**Published:** 2023-12-20

**Authors:** Cheng Huang, Haiyan Luo, Baitao Zeng, Chuanxin Feng, Jia Chen, Huizhen Yuan, Shuhui Huang, Bicheng Yang, Yongyi Zou, Yanqiu Liu

**Affiliations:** Department of Medical Genetics, Jiangxi Key Laboratory of Birth Defect Prevention and Control, Jiangxi Maternal and Child Health Hospital, Nanchang, Jiangxi, China

**Keywords:** *DYRK1A*, MRD7, novel variant, prenatal diagnosis, whole exome sequencing

## Abstract

**Background and purpose:** Intellectual disability-7 (MRD7) is a subtype disorder of intellectual disability (MRD) involving feeding difficulties, hypoactivity, and febrile seizures at an age of early onset, then progressive intellectual and physical development deterioration. We purposed to identify the underlying causative genetic factors of three individuals in each Chinese family who presented with symptoms of intellectual disability and facial dysmorphic features. We provided prenatal diagnosis for the three families and genetic counseling for the prevention of this disease.

**Methods:** We collected retrospective clinical diagnostic evidence for the three probands in our study, which included magnetic resonance imaging (MRI), computerized tomography (CT), electroencephalogram (EEG), and intelligence tests for the three probands in our study. Genetic investigation of the probands and their next of kin was performed by Trio-whole exome sequencing (WES). Sanger sequencing or quantitative PCR technologies were then used as the next step to verify the variants confirmed with Trio-WES for the three families. Moreover, we performed amniocentesis to explore the state of the three pathogenic variants in the fetuses by prenatal molecular genetic diagnosis at an appropriate gestational period for the three families.

**Results:** The three probands and one fetus were clinically diagnosed with microcephaly and exhibited intellectual developmental disability, postnatal feeding difficulties, and facial dysmorphic features. Combining probands’ clinical manifestations, Trio-WES uncovered the three heterozygous variants in *DYRK1A*: a novel variant exon3_exon4del p.(Gly4_Asn109del), a novel variant c.1159C>T p.(Gln387*), and a previously presented but rare pathogenic variant c.1309C>T p.(Arg437*) (NM_001396.5) in three families, respectively. In light of the updated American College of Medical Genetic and Genomics (ACMG) criterion, the variant of exon3_exon4del and c.1159C>T were both classified as likely pathogenic (PSV1+PM6), while c1309C>T was identified as pathogenic (PVS1+PS2_Moderate+PM2). Considering clinical features and molecular testimony, the three probands were confirmed diagnosed with MRD7. These three discovered variants were considered as the three causal mutations for MRD7. Prenatal diagnosis detected the heterozygous dominant variant of c.1159C>T p.(Gln387*) in one of the fetuses, indicating a significant probability of MRD7, subsequently the gestation was intervened by the parents’ determination and professional obstetrical operation. On the other side, prenatal molecular genetic testing revealed wild-type alleles in the other two fetuses, and their parents both decided to sustain the gestation.

**Conclusion:** We identified two novel and one rare mutation in *DYRK1A* which has broadened the spectrum of *DYRK1A* and provided evidence for the diagnosis of MRD7 at the molecular level. Besides, this study has supported the three families with MRD7 to determine the causative genetic factors efficiently and provide concise genetic counseling for the three families by using Trio-WES technology.

## Introduction

Intellectual disability-7 (MRD7 OMIM#614104) is caused by mutations in *DYRK1A,* which is an autosomal dominant hereditary disorder of autism and generally characterized by autism, intellectual disability (ID) features which cause primary microcephaly (−3 SD), nonverbal severe intellectual and physical disability, anxious behavior, and deformities. Facial abnormalities cover bilateral temporal stenosis, pointed nasal tips, large and underdeveloped ears, and deep-set eyes (van Bon et al., 2011). Early symptoms of MRD7 are feeding difficulties, febrile seizures, non-fluent motoric movements, and hypoactivity in infancy caused by central nervous system anomalies. The individual can develop other symptoms such as speech impairment, hypertonia, developmental delay, and autism behaviors, usually leading to the patient’s poor autonomy and adult social maladjustment. Less common features are known as optic nerve defects, cardiac anomalies, and contractures ataxia. Hitherto, there is no effective treatment strategy for MRD7, and receiving rehabilitation and anticonvulsant therapy can merely relieve the patients’ symptoms with MRD7, while poor compliance still remains a barrier.

The chromosome 21 at 21q22.13 positioned dual specificity tyrosine-phosphorylation-regulated kinase 1A (*DYRK1A* OMIM#600855), up to now, is the only known gene responsible for MRD7. *DYRK1A,* a gene function prominent in most of the characteristic features related to microcephaly, is highly conserved in the Down Syndrome critical region (DSCR). Loss-of-function mutations of *DYRK1A* can contribute to haploinsufficiency for the allelic gene leading to a decrease in surrounding ganglia about the soma and neurite complexity (Fotaki et al., 2002). Due to febrile seizures in fancy, presynaptic function in GABA releasing and re-uptaking and postsynaptic components’ function in GABA receptor trafficking and availability would transmit abnormally in GABAergic transmission (Kang et al., 2006; Qu and Leung, 2008). A primary diagnosis based on the clinical evidence included microcephaly, IUGR in the fetus period, intellectual disability, growth retardation, stereotype, facial gestalt, dysmorphic abnormalities, speech impairment, and clumsiness in fine motor patterns. As time goes on, with medical advances in various fields and the progress of DNA sequencing technologies, more and more *DYRK1A* variants have been identified in MRD7 patients, enabling further effective precise diagnosis for genetic confirmation. In 2008, *DYRK1A* was verified firstly as a factitive gene for MRD7 (Matsumoto et al., 1997; Møller et al., 2008). On the basis of the online data library Orphanet Reports Series (https://www.orpha.net), the MRD7 prevalence rate caused by a *DYRK1A* point mutation is less than 1/1000000. To date, 68 pathogenic variants in *DYRK1A* have been presented of this disease. There have been almost 200 mutations in *DYRK1A* presented, besides, a totality of 191 variants in *DYRK1A* have been presented and more than 80 variants resulting in MRD7 have been reported, based on the data library professional Human Gene Mutation Database (HGMD). Besides, further extensive explorations on the spectrum of *DYRK1A* resulting MRD7 are necessary, due to the diverse and large population condition of China. Hitherto, in China, only two reports on individuals were confirmed with MRD7 (Qiao et al., 2019; Ma et al., 2021).

In this study, using Trio-whole exome sequencing (WES), we confirmed the genetic incentive factors that two novel mutations and one rare mutation in three Chinese families of MRD7. Besides, for the prevention of MRD7 in these cases, we presented ethical issues concerning genetic counseling, such as prenatal diagnosis.

### Clinical presentations

**FIGURE 1:**
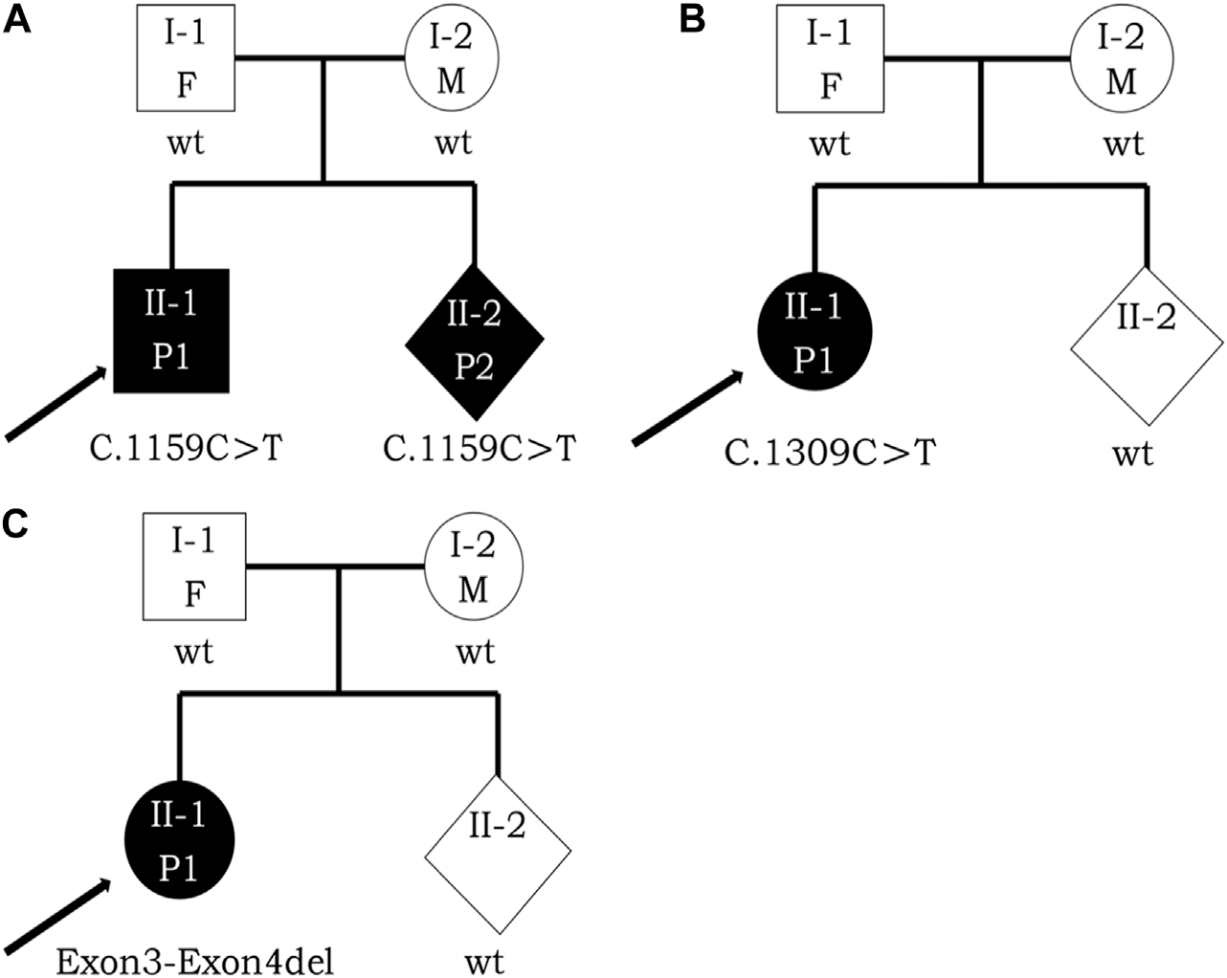
Pedigree analysis of these three families with *DYRK1A* variants. Opening symbols, unaffected; filled symbols, affected; squares, male; rounds, female; rhombus, fetus with unknown gender; arrowheads, the proband; F, father; M, mother; P1, patient 1; P2, patient 2; wt, wild type. Three black arrowheads represent the probands [**(A)**, II-1, II-2; **(B)**, II-1; **(C)**, II-1) respectively. **(A)** I-1, proband’s father, 36 years old; I-2, proband’s mother, 30 years old; II-1, the proband, 5 years old; II-2, the fetus at the gestational age of 16 + 3 weeks. **(B)** I-1, proband’s father, 29 years old; I-2, proband’s mother, 29 years old; II-1, the proband, 4 years old; II-2, the fetus at the gestational age of 16 + 5 weeks. **(C)** I-1, proband’s father, 28 years old; I-2, proband’s mother, 26 years old; II-1, the proband, 4 years old; II-2, the fetus at the gestational age of 18 + 5 weeks.

In Jiangxi Maternal and Child Hospital (Jiangxi, China), the three probands from three respective nonconsanguineous healthy Chinese families were admitted to the medical genetics department and pediatric department. The three individuals’ clinical features included intellectual and physical disability and special facial features. Their families’ history had no evidence of skeletal, intellectual, neurological, metabolic, developmental, or any other heritable diseases. These three individuals were born with varying degrees of intrauterine growth restriction (IUGR) and microcephaly. Feeding difficulties, febrile seizures, and hypotonia were noticed during the infantile period, representing the typical facial gestalt in childhood, and all of them did not reach the normal intellectual development milestones. Besides, the brain CT, MRI, ECG examination, clinical investigations including intelligence testing, chromosomal karyotype analysis, serum amino acid determination, blood ammonia, and succinyl acetone level testing extracted in peripheral blood at 2.5 years old were conducted for the proband with exon3_exon4del variant in *DYRK1A*. Results of intelligence testing indicated intellectual disability (ID) in terms of social activity, language, fine motor skills, and sport. Analysis of serum amino acid determination indicated that the leucine and valine were moderately higher than normal levels. CT and MRI revealed bilaterally enlarged ventricles in the proband, while ECG showed no significant abnormalities. This proband also demonstrated the dysmorphic features of MRD7 including deep-set eyes, pointed nasal tip, and thin upper lip. Since the three probands’ mothers were in the process of gestation, they hoped to give birth to a healthy child. Thus, for professional prenatal genetic counseling, they sought guidance from the Medical Genetics Department. The three family trees are presented in Figure 1. The Ethics Committee of Jiangxi Maternal and Child Health Hospital authorized this study to utilize the clinical information and collection of samples, and informed consent forms were received from the patients.

## Methods

### Trio-WES

An amniotic fluid aspiration sample was adopted by amniocentesis and peripheral blood was collected from every participant after obtaining informed written consent or participation. Genomic DNA was collected from leukocytes, following manufacturers’ instructions. For analyzing and measuring participants’ DNA, we performed the NanoDrop spectrophotometer from Thermo Scientific in Wilmington, DE, United States. Above all, to formulate the WES libraries, 3 µg of participants’ genomic DNA clipped by performing ultrasonication (Covaris S220 Ultrasonicator). Next, by using the BGI V4 chip, we captured and concentrated the labeled regions containing exons and splicing sites of more than 20,000 genes in the human genome, after that, based on the manufacturer’s protocol, we implemented the sequencing by the MGISEQ-2000 platform (BGI, Shenzhen, China) with the pair-end sequencing. The quality control index of sequencing data met demand as follows: the average coverage depth for the labeled area was ≥180×, and the ratio of sites with an average depth >20× of the labeled regions was >95%. By applying the BWA to remove duplications, the sequenced parts were arrayed to the UCSC RefSeq database hg19 human reference genome. Then the GATK standard genotype tool was performed for variant searching of single nucleotide variations, insertions, or deletions (Indels) and copy number variations at the exons’ level using Exome Depth according to the algorithms based on probability and quality. Concisely, in the process, we filtered out the general pleomorphisms as dbSNP (minor allele frequency>0.01), 1,000 genomes (genotype frequency>0.005), gnomAD, EVC, and synonymous single nucleotide variants.

### Variants annotations and interpretations

The gene nomenclature abides by the traditional patterns of the Human Genome Organization Gene Nomenclature Committee; the variant nomenclature abides by the traditional patterns of the Human Genome Variation Society and International Union of Pure and Applied Chemistry. Variant annotations and screenings were based on clinical phenotypes of the subject, population databanks (dbSNP, ExAC, 1,000 Genome), disease databanks (OMIM, HGMD, SMART, Clinvar), and biological resource prediction tools (SIFT, Mutation Taster, and Polyphen2). On the basis of the criteria of the American College of Medical Genetics and Genomics (ACMG) and the American Society for Molecular Pathology, interpretations of pathogenicity for variants were formulated. Detailed interpretations of the guidelines were consulted by the ClinGen Sequence Variation Interpretation Working Group and the British Society of Clinical Genomics (ACGS). When a novel/rare variant predicted to be pathogenic was identified in a sole acknowledged intellectual disability gene and possessed with the parallel pattern of inheritance, it should be deemed as a prospective candidate variant.

### Confirmation of the three variants by Sanger sequencing and quantitative PCR

For the sake of validation about all the exons’ sequencing consequences and segregations’ analytical results, Sanger sequencing was operated by 3500DX Genetic Analyzer (Applied Biosystems) to verify the candidates for the two families with the point mutations, while quantitative PCR (qPCR) was performed on the family with exons deletion of cDNA using the QuantStudio 5 (Applied Biosystems). With normalized reference sequences and visualizations, the sequence analysis was performed by Seqman software (Technelysium, South Brisbane, QLD, Australia). Based on the online databank of the University of California, Santa Cruz (UCSC), we designed the reference sequences. Table 1 lists the four individual primer pairs concepted for verifying the three mutations.

**TABLE 1:** Confirmation of the four primers purposed for the qPCR/Sanger sequencing of cDNA.

Primers’ names	Sequencings (5′→ 3′)	Products’ lengths (bp)
DYRK1A qE3F	CCT​TCA​TCT​GTT​CGG​CTT​GC	99
DYRK1A qE3R	GCG​ACG​GTC​ACT​GTA​CTG​AT	
DYRK1A qE4F	ACA​TGC​AGG​TTA​CAG​AAG​AGG​G	118
DYRK1A qE4R	GGA​AGG​TTT​GGG​GCA​TCC​G	
DYRK1A E8F	TGA​GCA​GGA​GTA​GAT​GTA​CAG​T	400
DYRK1A E8R	CCA​TTA​ATC​AAA​CAC​TGG​TCC​AT	
DYRK1A E9F	CAT​AGT​TTA​CTG​ACC​CCT​GGG​C	724
DYRK1A E9R	AAA​TAT​TGG​AGC​TTT​GGG​GGA​GA	

### Prenatal diagnosis of the fetuses

For the three probands following the three factitive variants of *DYRK1A* were confirmed, those families expressed their anticipation to carry out the prenatal diagnosis for their fetuses at a fitted gestational period. Amniotic fluid samples in the sum of 20 mL were obtained at the gestational age of 16W + 3, 16W + 5, and 18W + 5 respectively from each pregnant mother’s uterine cavity from the three families following the B ultrasonography guidance. By using the QIAamp DNA Mini Kit (Qiagen, Germany), the extraction of fetal DNA from the amniotic fluid was implemented, according to the protocols of the manufacturer. In order to explore the mutations’ state of c.1159C>T and c.1309C>T by applying the forward and reverse sequencings, exon3_exon4del in *DYRK1A* was analyzed as presented before. Simultaneously, the matrilinear cell pollution has been removed by operating the quantitative fluorescent polymerase chain reaction, in light of the instruction presented before (Noveski et al., 2019).

### Statistical analysis

The statistical analysis was performed using SPSS 20.0 software.

## Results

Based on clinical symptoms and genetic discoveries, the three probands were diagnosed with MRD7. One of the fetuses was genetically diagnosed with MRD7 while the other two showed wild-type alleles in the detected locus by prenatal diagnosis.

### Whole exome sequencing findings of patients’ phenotypes-related variants and Sanger sequencing validation

By using Trio-WES, the raw data sequencing production for each candidate approached at least 23,170.74 Mb. With the mean of 273.8.85×sequencing depth, to the human genome hg19, approximately 99.72% of sequencing reads were arranged. It is noteworthy that, in the three probands, the three heterozygous disorder-related variants in *DYRK1A* (NM_001396.5), 2 novel variants including c. 1159C>T and exon3_exon4del, and a rare variant c.1309C>T, passed the screening standards. About the three variants, in exon3 and exon4, variant exon3_exon4del gives rise to deletion of protein translation from residue 4 to residue 109 p.(Gly4_Asn109del), and the two variants c.1159C>T and c.1309C>T lead to the prophase termination in protein translation period at residue 387 p.(Gln387*) and residue 437 p.(Arg437*) by inducing a premature stop codon respectively. Furthermore, we collected and analyzed the incidental findings of other genes in insufficient pathogenic evidence for the three probands, which was placed in (Supplementary Table S1). Subsequently, Sanger sequencing and qPCR validated these three mutations in the three families.

### Variant annotations and interpretations according to the ACMG guidelines

**FIGURE 2:**
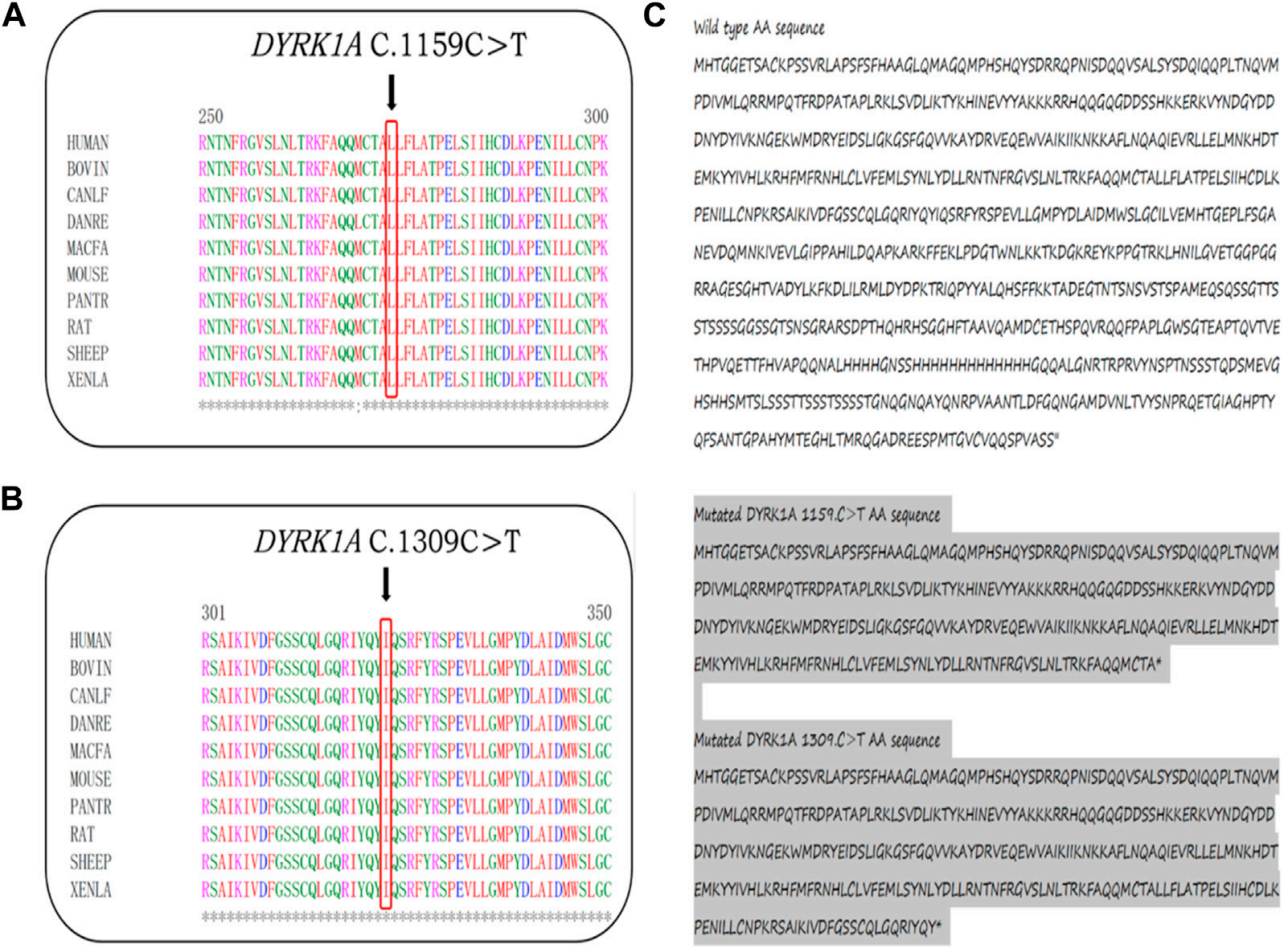
**(A,B)** The truncating amino acids’ location, pointed by black arrows, among species are highly evolutionarily conserved. **(C)** At the 387th and 437th of *DYRK1A* predicted premature termination codon.

**FIGURE 3:**
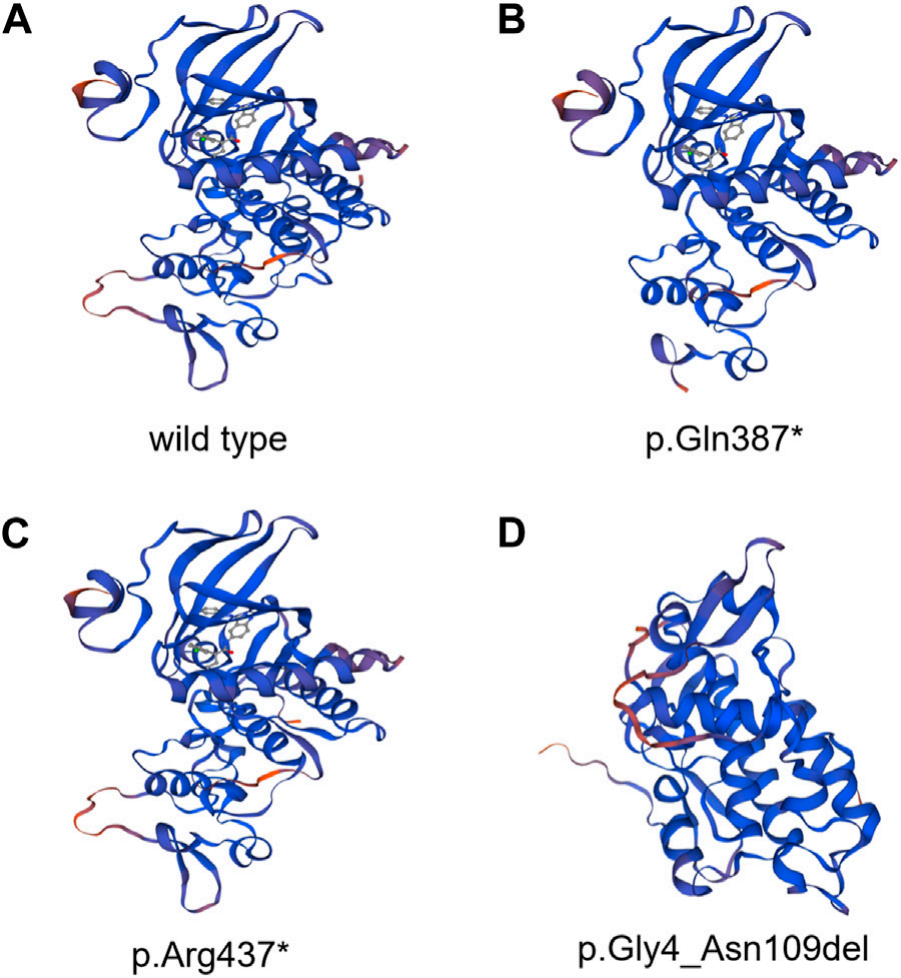
DYRK1A protein of homologous modeling three-dimensional structures by Swiss-model. **(A)** Whole structure of wild-type DYRK1A protein. **(B)** DYRK1A protein of p.Gln387* (c.1159C>T) mutation. **(C)** DYRK1A protein of p.Arg437* (c.1309C>T) mutation. **(D)** DYRK1A protein of p.Gly4_Asn109del (exon3_exon4del) mutation.

In the 1,000 Genomes databank the variant c. 1159C>T and exon3_exon4del were not searched, HGMD master databank, Genome Aggregation Database (gnomAD), nor in the Exome Variant Server. On the flip side, in our 200 healthy control cohorts, the two variants were not found. The variant exon3_exon4del is predicted to result in the 105-amino-acid-deletion from the 4th residue to the 109th residue in exon3 and exon4, whereas the variant c.1159C>T is speculated to produce a premature stop codon introduction by a substitution of glutamine that lead to a truncating protein at the 387th amino acid in DYRK1A. The DYRK1A protein family members’ alignments were revealed By the ClustalX online software that amino acids of DYRK1A from the 4th residue to the 387th amino acid are highly evolutionarily conserved, which is displayed in Figure 2. We predicted the the deleted protein structure resulted from two novel mutations and a premature truncation could be produced via selective degradation by the nonsense-mediated mRNA decay, respectively. By using algorithms of multiple bioinformatic methods which include SIFT (sift.jcvi.org), Mutation Taster (www.mutationtaster.org), and polyphen-2 (genetics.bwh.harvard.edu/pph2), these two novel variants were calculated to be deleterious. We build the homologous modeling three dimension structure by Swiss-model (SWISS-MODEL (expasy.org)) of wild type (Seq Identity: 100.00%, QMQE: 0.92, QMEAN: 0.87 ± 0.05), p.Gln387* (c.1159C>T) mutation (Seq Identity: 100.00%, QMQE: 0.92, QMEAN: 0.88 ± 0.05), p.Arg437* (c.1309C>T) mutation (Seq Identity: 100.00%, QMQE: 0.92, QMEAN: 0.87 ± 0.05) and p.Gly4_Asn109del (exon3_exon4del) mutation (Seq Identity: 99.60%, QMQE: 0.91, QMEAN: 0.87 ± 0.05) placed in Figure 3 (Camacho et al., 2009; Soundararajan et al., 2013; Bertoni et al., 2017; Bienert et al., 2017; Waterhouse et al., 2018; Steinegger et al., 2019; Studer et al., 2020; Studer et al., 2021).

On the basis of the latest ACMG guideline, the two novel variants in our study were consequently classified as likely pathogenic (PSV1+PM6). Furthermore, we found the identified truncating variant c1309C>T inducing the alteration of the 437th Arg into a stop codon. This variant is considered pathogenic (PVS1+PS2_Moderate+PM2) in light of the ACMG guidelines. Overall, considering the clinical manifestations and examinations, the diagnosis of MRD7 was validated by the genetic analysis in the three families.

### Genetic findings for the three fetuses by amniocentesis

**FIGURE 4:**
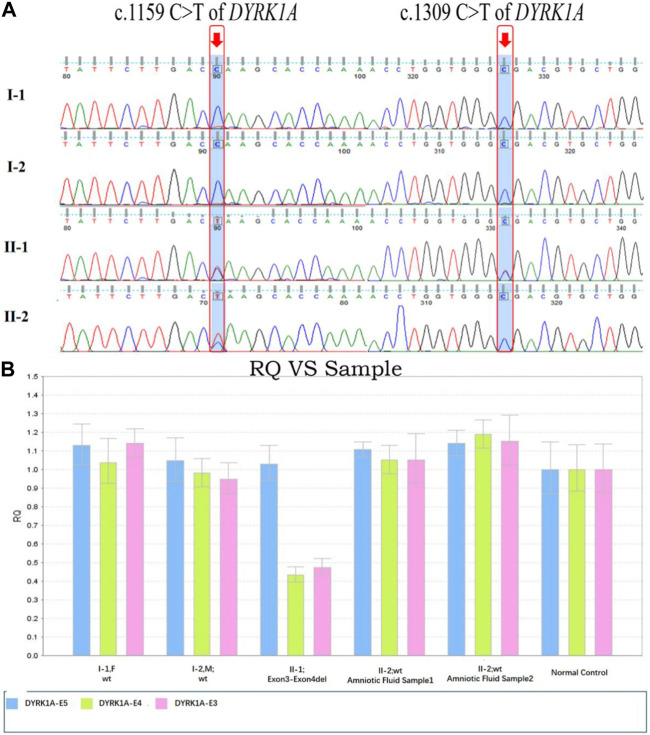
*DYRK1A* variants sequence consequences and variants analysis in distinct domains of *DYRK1A*. **(A)** The c.1159C>T and c.1309C>T variants of *DYRK1A* sequence chromatograms were identified for the two families’ members. **(B)** Fluorescence quantitative polymerase chain reaction (qPCR) bar chart for the exon3-exon4del variant of the *DYRK1A* detected in the family members, compared with normal control. Blue regions represent exon3, green regions represent exon4, and pink regions represent exon5.

Amniotic fluid analysis was produced after an additional 7 days, which identified one of the three fetuses was heterozygous autosomal dominant for c. 1159C>T possessing the equal genetic variant in their family as the proband. The other two fetuses were detected with wild-type alleles within the c.1309 and exon3_exon4 locus. In Figure 4, the chromatograms of Sanger sequence analysis and quantitative polymerase chain reaction testing for the members of the three families are displayed respectively. After prudent and adequate consideration, the first family resolved to intervene in the gestation at the earliest possible time and the other two families chose to continue the gestation. For these two families, subsequent interval follow-ups showed no anomaly in the two born children.

## Discussion

**FIGURE 5:**
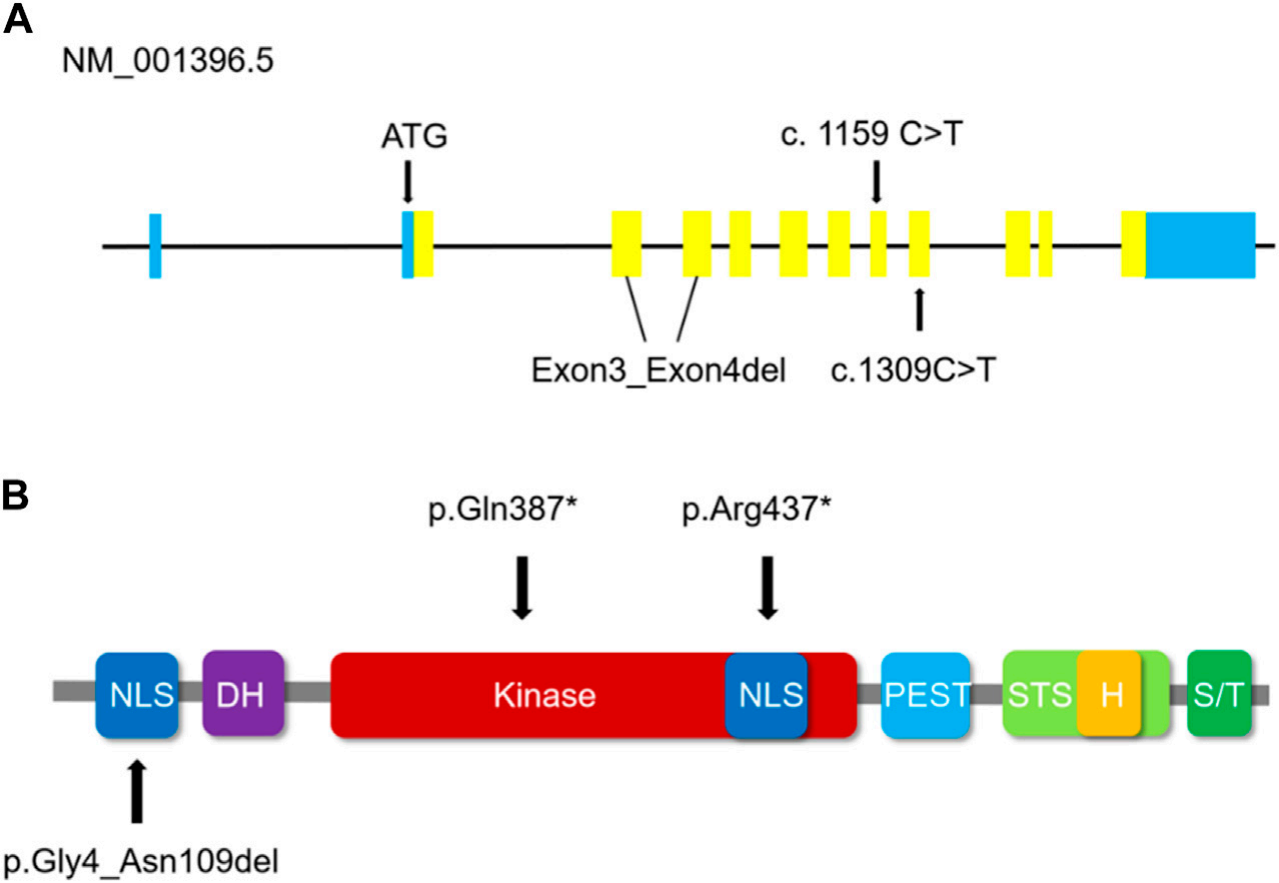
**(A)** Schematic representation of *DYRK1A* (NM_001396.5) coding regions illustrates the positions of the three variants in this study. Blue regions represent non-coding regions, yellow regions represent the coding sequence. **(B)** The domains in which the validated *DYRK1A* variants are positioned. The variant exon3_exon4del has an impact on the first NLS domain of the *DYRK1A* protein. The truncating amino acids alteration p.(Gln387*) of the c.1159 C>T variant and p.(Arg437*) of the c.1309 C>T variant are positioned in the Kinase domain and the second NLS domain respectively of the *DYRK1A* protein. NLS indicates the nuclear localization signals in the dark blue domain. DH indicates the *DYRK* homology box in the purple domain. STS indicates the speckle targeting signal in the light green domain. H indicates the histidine repeat in the yellow domain, and S/T indicates the serine/threonine repeat in the dark green domain. Kinase indicates protein kinase in the red domain, and the PEST is concentrated in proline, glutamic acids, serine, and threonine in the light blue domain.

*DYRK1A* encodes a member of the dual specificity tyrosine-phosphorylation-regulated kinase (DYRK) family, a 58 kDa tyrosine-phosphorylation-regulated kinase possessing with serine/threonine and tyrosine kinase activities. Besides, on chromosome 21 *DYRK1A* resides in the Down syndrome critical region (DSCR) and is reckoned responsible for intellectual disabilities as a mighty housekeeping gene related to Down syndrome (Guimerá et al., 1996; Matsumoto et al., 1997; Eisenberg and Levanon, 2013). Of the five mammalian DYRKs, which is the highly conserved DYRK family, the *DYRK1A* regulates neuronal morphogenesis by phosphorylating cytoskeletal elements and also participates in the development of olfactory, visual, and central nerve systems. *DYRK1A* includes a conserved catalytic kinase domain prior to a distinctive DYRK homology (DH) box, two nuclear localization signals (NLS), one preceding and the other one with the kinase domain, a PEST domain, a speckle targeting signal (STS), a histidine repeat and a serine/threonine repeat (Evers et al., 2017). The guardian-like functional DYRK-homology box (DH box) stabilizes the kinase domain in a middling structure during the process of protein maturation with tyrosine autophosphorylation activity (Kinstrie et al., 2010), as shown in Figure 5. The NLS is discerned by importin protein in the cytosol, which could conduct the transfer of the protein to the nuclear pores, while the PEST indicates the signal sequence of degradation, a characteristic of short-lived proteins. Within the nucleus, the histidine repeat region labels the protein to bonding factor assembly (Alvarez et al., 2003). *DYRK1A* is expressed in neuroepithelia precursor cells during the prophase of embryonic development in *Drosophila*, which sets the conversion step from proliferations to neurogenic divisions, and mutations of *DYRK1A* could induce a microcephaly phenotype and abnormal retinal forming progression (Tejedor et al., 1995; Fotaki et al., 2002; Hämmerle et al., 2002; Laguna et al., 2008; Brault et al., 2021). In our case, the three mutations were confirmed to the family co-segregation by Sanger sequencing and fluorescence quantitative polymerase chain reaction. We analyzed the homologous alignment of protein sequences, which reveals that the two variants and one sequence are highly conserved between humans and other species. DYRK1A protein constructed using Swiss-model homologous modeling software, the three-dimensional structure of wild type differs from variant forms placed in Figure 3. c.1159C>T and c.1309C>T lead to two premature stop codons at residue 387 and residue 437 causing the termination of the primary structures of DYRK1A proteins. The nonsense variant c.1159C>T and c.1309C>T are located in exon8 and exon9 respectively, which is the interior of the kinase domain and lead to the elimination of the helix-turn-helix functional component, disrupting the critical activation segment. The variant exon3_exon4del is located in the NLS domain, which could interfere with the transport of proteins into the nucleus. The nonsense variant c.1159C>T and c.1309C>T variant lead to the elimination of and deletion of inframe shift exon3_exon4del interfere with the nuclear transport, and then the abnormal assembly of helix-turn-helix functional component and NLS domain, which damages the stability and flexibility of DYRK1A protein structure ultimately affect the function of the tyrosine autophosphorylation activity. Based on the DYRK1A protein prediction models of wild type, the variant forms cannot complete the protein structure due to the nonsense mutations or inframe shift, leading to an early end and deletion of the translation process, which has a significant impact on protein function. It has been reported that haploinsufficiency of *DYRK1A* is related to intellectual disability (ID), autism spectrum disorder (ASD), intrauterine growth restriction (IUGR), and development delay (DD) (van Bon et al., 2016; Earl et al., 2017; Arbones et al., 2019; Morison et al., 2022).

Intellectual disability-7 was first reported in 1997, and MRD7 patients often show microcephaly, severe intellectual disability, dysmorphic features and feeding difficulties, and febrile seizures in childhood (Matsumoto et al., 1997; Møller et al., 2008). Other clinical manifestations may include intrauterine growth restriction, speech, anxious autistic behavior, and hand stereotypies. These symptoms mentioned above often lead to neurodevelopmental dysplasia (Widowati et al., 2018; Arbones et al., 2019; Mewasingh et al., 2020). The majority of patients with MRD7 present the imaging feature of diffuse cortical atrophy on MRI at a relatively early age (Courcet et al., 2012). In a large series of 31 MRD7 patients as listed in Table 2, although microcephaly (96.7%), DD (96.7%), and ID (96.7%) are the most general features of this disease, there is still a phenotype variability, with other general clinical discoveries like seizures (90%), feeding problems (83.4%), facial gestalt (80%), short stature (50%), IUGR (46.7%), ASD (40%), gait disturbance (26.7%), sleep disturbance (13.4%), hypertonia (13.4%) and dental anomalies (3.3%). Besides, there are uncommon clinical features but severe in significant organs of this disease including eye abnormalities (33.3%), musculoskeletal features (16.7%), cardiac defects (6.7%), and gastrointestinal problems (6.7%). However, it is notable that the probands in our cases did not show evidence of eye anomalies, cardiac defects, or gastrointestinal problems. As placed in (Supplementary Table S1), in the incidental findings of insufficient pathogenic evidence genes for the three probands by trio-WES, we found the c.-115C>G and c.*286A>T variants of *TUBB3* in AD heterozygote inherited pattern estimated as VUS for the proband of c.1159C>T, c.194C>T and c.874A>G variants of *NALCN* in AD heterozygote inherited pattern estimated as VUS for the proband of c.1309C>T, 16p12.2del variants of *C16orf52*, *CDR2*, *EF2K*, *POLR3E*, *VWA3A*, and *UQCRC2* in heterozygote inherited pattern estimated as pathogenic for the proband of exon3_exon4del, which have the related symptoms about neurological and psychiatric developmental abnormalities to MRD7. The c.-115C>G and c.*286A>T variants of *TUBB3* could give rise to the dysfunction of neuronal-specific β-tubulin isotype III, which may lead to compound cortical dysplasia with other brain developmental abnormalities type 1 associated with the syndrome of MRD7, however, the location of *TUBB3* variant is in the non-coding region and comprehensively estimated as LP. The c.194C>T and c.874A>G variants of *NALCN* were inherited from proband’s father and were estimated as VUS, her father did not show the symptoms of the same variants of *NALCN* and the certain variants’ pathogenic proof were not retrieved in the database presented as before (Radwitz et al., 2022; Puri et al., 2023). The 16p12.2del variants were inherited from the proband’s mother, which was estimated as pathogenic for her mother’s corresponding symptoms. Furthermore, the variant in *UQCRC2* is related to Mitochondrial complex III deficiency, nuclear type 5, which is a mitochondrial respiratory chain disorder characterized by highly variable clinical phenotypes including mitochondrial encephalopathy, psychomotor delay, ataxia, severe dysplasia, liver dysfunction, metabolic acidosis, renal tubular disease, muscle weakness and exercise intolerance (Hernando et al., 2002; Ballif et al., 2007; Bansept et al., 2023). For the proband who possesses the mutations of *DYRK1A* and *UQCRC2* prescribed as before, the symptoms caused by these two gene mutations have a certain degree of similarity, while the missing fragments caused by 16p12.2del are relatively functional, therefore we estimated this mutation as pathogenic. However, the common symptoms of her mother include ID and DD, but did not show any evidence of the microcephaly, seizures, feeding difficulties, and facial gestalt, which were diagnosed in the proband of exon3_exon4del. The experimental validation of *DYRK1A* for the three probands’ mutations requires we conduct further research.

**TABLE 2:** *DYRK1A* mutations screening and clinical manifestations of patients.

Cases	Patients	Sex/age (years/weeks)	Genotype/mutation and amino acid/functional change	Brain MRI/CT	Clinical features	Related PMID
This study	Patient 1	F	c.1159C>T; p.Gln387*	Enlarged ventricles, and brain atrophy	Microcephaly, ID, DD, seizures, and facial gestalt	
		5 years				
	Patient 2	Unknown	c.1159C>T; p.Gln387*	Brain atrophy	Abnormal plantar posture, IUGR, and microcephaly	
		18+ weeks				
	Patient 3	M	c.1309C>T; p.Arg437*	hypoplastic corpus callosum	Microcephaly, ID, DD, and seizures feeding difficult, and facial gestalt	
		4 years				
	Patient 4	M	exon3-exon4del; p.Gly4_Asn109del	Enlarged bilateral ventricles	Microcephaly, ID, DD, and seizures feeding difficulties, and facial gestalt	
		4 years				
Case1	Patient1	M	c.367C>T; p.Gln123*	Normal	Albinism, microcephaly, IUGR, mild-moderate ID, DD, feeding difficulties, seizures, hypotonia, facial gestalt, abnormal gait, high arch feet, long hallux, small hands, sleep disturbances, and astigmatism	25707398
		32 years				
	Patient2	F	c799C>T; p.Gln267*	N/A	Cardiac murmur, aortic valve stenosis, microcephaly, severe ID, DD, seizures, short stature, hypotonia, abnormal gait, Spinal deformity, contractures, kyphosis, feeding difficulties, and facial gestalt	
		59 years				
	Patient3	M	c.1491del; p.Ala498Profs*94	Normal	microcephaly, mild ID, DD, ASD, seizures, sleep disturbance, and feeding difficulties	
		10 years				
	Patient4	M	c.143_144del; p.Ile48Lysfs*2	N/A	Mild cerebral palsy, microcephaly, mild ID, DD, ASD, seizures, abnormal gait, spinal deformity, arachnodactyly, feeding difficulties, facial gestalt, partial cutaneous syndactyly toes, arachnodactyly, and sleep disturbances	
		12 years				
	Patient5	F	c.665-9_665-5delTTCTC; p.fs*22	Normal	Microcephaly, mild-moderate ID, DD, ASD, seizures, feeding difficulties, facial gestalt, high arch feet, long hallux, and delayed primary dentition	
		10 years				
	Patient6	M	c.516 + 2T>C; /	Myelination delay, hypoplastic corpus callosum, and brain atrophy	Bilateral tibial osteochondrosis and exostoses, microcephaly, severe ID, DD, ASD, seizures, hypotonia, spinal deformity, interdigital webbing, partial cutaneous syndactyly2-4 toes, short fifth toe, hallux vagus, feeding difficulties, and facial gestalt	
		16 years				
	Patient7	F	c.1098 + 1G>A; /	Normal	Microcephaly, severe ID, DD, ASD, seizures, hypertonia, abnormal gait, caval varus foot deformity, supernumerary teeth, and feeding difficulties	
		17 years				
	Patient8	M	c.208-1G>A; p.Val70*	N/A	Asperger’s disorder during childhood, microcephaly, ID, DD, ASD, seizures, short stature, sleep disturbance, feeding difficulties, and facial gestalt	
		18 years				
	Patient9	M	c.1240-2A>G; p.Glu414Valfs*76	Normal	Microcephaly, moderate ID, DD, ASD, seizures, short stature, hypertonia, spinal deformity, Partial cutaneous syndactyly2-4 toes, short fifth toe, feeding difficulties, and facial gestalt	
		23 years				
Case2	Patient1	F	c.1309C>T; p.Arg437*	Thin optic nerves	Microcephaly, ID, DD, seizures, short stature, deep-set eyes, ears deformity, thin upper lip, clumsy, and feeding difficult	25920557
		6.5 years				
	Patient2	M	c.763C>T; p.Arg255*	Enlarged ventricles, hypoplastic corpus callosum	Microcephaly, IUGR, moderate ID, DD, ASD, seizures, ametropia, deep-set eyes, pointed nasal tip, micrognathia, feeding difficult, and facial gestalt	
		11 years				
	Patient3	M	c.613C>T; p.Arg205*	absent	Microcephaly, IUGR, ID, DD, ametropia, deep-set eyes, ears deformity, pointed nasal tip, thin upper lip, micrognathia, feeding difficult	
		15 years				
	Patient4	M	c.1036T>C; p.Ser346Pro	N/A	Microcephaly, IUGR, ID, DD, ASD, seizures, deep-set eyes, ametropia, ear deformity, pointed nasal tip, thin upper lip, micrognathia, clumsy, feeding difficult, facial gestalt	
		3.5 years				
	Patient5	F	c.1763C>A; p.Thr588Asn	N/A	Microcephaly, IUGR, ID, DD, ASD, seizures, short stature, deep-set eyes, thin upper lip, micrognathia, clumsy, facial gestalt	
		32 years				
	Patient6	M	c.844dupA; p.Ser282Lysfs6	Enlarged ventricles	Microcephaly, ID, DD, seizures, deep-set eyes, ear deformity, pointed nasal tip, thin upper lip, micrognathia, feeding difficulties, facial gestalt	
		3 years				
	Patient7	M	c.621_624delinsGAA; p.Glu208Asnfs3	Mildly enlarged ventricles	Microcephaly, ID, DD, deep-set eyes, ametropia, pointed nasal tip, thin upper lip, micrognathia, feeding difficulties, facial gestalt	
		4 years				
	Patient8	F	c.1232dupG; p.Arg413Thrfs10	Enlarged ventricles, cerebellar atrophy	Microcephaly, IUGR, ID, DD, seizures, short stature, deep-set eyes, ear deformity, pointed nasal tip, thin upper lip, micrognathia, clumsy, feeding difficult, facial gestalt	
		5 years				
	Patient9	M	c.945dupG; p.Gln316Alafs24	Mildly enlarged ventricles	Microcephaly, ID, DD, ASD, seizures, short stature, ametropia, ear deformity, thin upper lip, clumsy, feeding difficult, facial gestalt	
		6 years				
Case3	Patient1	M	c.613C>T; p.Arg205*	Enlarged lateral ventricles, cortical atrophy	Microcephaly, mildly ID, DD, seizures, enophthalmia, micro retrognathia, smooth philtrum, thin upper lip, syndactyly, large ears, frontal bossing, low columella, micropenis, bilateral inguinal hernias, feeding difficulties, and facial gestalt	25641759
		5 years				
	Patient2	M	c.932C>T; p.Ser311Phe	Enlarged lateral ventricles, cortical atrophy	IUGR, single umbilical artery, moderately ID, DD, seizures, large ears, small mouth, long, philtrum, asthma, numerous otitis media, and facial gestalt	
		3 years				
Case4	Patient1	M	T (9; 21) (p12; q22); /	Corpus callosum hypoplasia	IUGR, microcephaly, mildly ID, DD, seizures, stereotypical behavior, short stature, abnormal gait, inguinal hernia, hypermetropia, feeding difficulties, and facial gestalt	18,405,873
		2 years				
	Patient2	F	T (2; 21) (q22; 22); /	N/A	IUGR, microcephaly, severe ID, DD, seizures, spinal deformity, short fifth toe, ventricle spectrum defect, aortic valve insufficiency, feeding difficulties, and facial gestalt	
		13 years				
Case5	Patient1	F	Exon9-exon11del; /	Brain atrophy	IUGR, microcephaly, severe ID, DD, ASD, seizures, abnormal gait, hallux valgus, short fifth toe, breast aplasia, feeding difficulties, and facial gestalt	21294719
		37 years				
Case6	Patient1	F	Exon1del; /	Normal	IUGR, microcephaly, global ID, DD, seizures, short stature, hypertonia, short stature, abnormal gait, feeding difficulties, and facial gestalt	23099646
		4 years				
	Patient2	F	c.290_291delCT; p.Ser97Cysfs*98	Brain atrophy, and enlarged ventricles	IUGR, microcephaly, moderate ID, DD, seizures, feeding difficulties, and facial gestalt	
		14 years				
Case7	Patient1	M	c.930C>A; p.Tyr310*	mild prominence of lateral ventricles, enlarged peri-cerebral spaces, high palate, delayed myelination, and a thin corpus callosum	IUGR, microcephaly, micrognathia, ID, DD, seizures, abnormal gait, hypertonia, short stature, deep-set eyes, large ears, pointed nasal tip, downturned mouth, and micrognathia	31803247
		4 years				

CT, computerized tomography; DD, developmental delay; IUGR, intrauterine growth restriction; ID, intellectual delay; MRI, magnetic resonance imaging; N/A, not available; F, female; M, male.

To date, the majority of reported MRD7 patients have been sporadic cases resulting from a *de novo* pathogenic variant of the *DYRK1A* gene (Hamdan et al., 2014). With accordingly negligible risks of recurrence, apparent *de novo* mutations have been reckoned as the primary occurrences for germline or zygotic incidents (Xu et al., 2023). Nevertheless, for the pathogenic variant, those with a high ratio are truly inherited from a parent mosaic of which there is little evidence to prove. In our study, the fetus was detected with a heterozygous pathogenic variant of c.1159C>T p.(Gln387*) for *DYRK1A*, the same as the proband, which indicates a high probability of parental mosaicism by the occurrences of a same *de novo* mutation twice in a family. For a family with a childbearing history induced by an obvious *de novo* pathogenic variant affected by a genetic disease, the indetermination about the existence and the proportion of parental mosaicism gives cause counseling on the risk of recurrence to be imprecise and challenging. Furthermore, the risk of recurrence relies on whether the parental mosaic mutation exists in the paternal or maternal germ cell system and on the mutated reproductive cells’ harboring ratio (Campbell et al., 2014). It has been known that Sanger sequencing and the other conventional molecular technologies are limited in identifying the levels of somatic cell mosaicism for variant allele fraction or variant allele frequency (VAF) higher than 10%–20%. The normative exome sequencing variant-calling pipelining cannot routinely detect the existence of VAF at less than 10% level (Gambin et al., 2020). Nevertheless, in recent years progress in genomic technologies has improved the detection capability to be able to identify and analyze mosaicism at a low level (Pareja et al., 2022). Quantitative polymerase chain reaction (qPCR), high-depth sequencing, droplet digital PCR (ddPCR), single-base extension assays, and Multiple Independent Primer PCR Sequencing (MIPP-seq) all already proved efficient in improving the identification and quantification for mosaicism of low-level (Uchiyama et al., 2016; Doan et al., 2021; Zemet et al., 2022). In this study, due to the limitation of local technology and the burden of economic spending for the family of parent mosaicism detection, this family refused to detect the parent mosaicism. We respected their choices and informed them of the genetic cause of this situation.

Nowadays, diagnosis of MRD7 is generally established on the integration of typical clinical features and genetic analysis. Since *DYRK1A* was first defined as the inducing gene for MRD7 by Moller in 2008, clinical testing such as the ultrasonic evaluation of intrauterine fetal growth, and neurological, psychiatric, and auxiliary examinations was promoted and has helped a lot in the clinical diagnosis of MRD7 (Wong, 2009; Edwards and Hui, 2018; Davidson et al., 2021; Masselli et al., 2021). In recent years, WES has speedily developed as the most popular targeted enrichment method, which is peculiarly used to operate for monogenic (“Mendelian”) disorders in genetic testing at the molecular level. The exome includes about 85% of mutations with vast effects on disease-related traits, and shares only nearly 2% human genome. In our described cases, Trio-WES was performed for the unbiased analysis of the whole genes, time-efficient to sift the candidate genes preceding to detecting and reduced the demand for an economic-consuming (Singleton, 2011; Petrovski et al., 2019). According to the integration of Trio-WES and clinical background, the diagnosis pattern of MRD7 has been promoted enhancing the symptomatic diagnosis, prenatal diagnosis, and genetic counseling.

## Conclusion

We presented two novel likely pathogenic variants and one rare likely pathogenic variant inducing heterozygous presentations of *DYRK1A-*associated MRD7 and described the clinical heterogeneity for those affected populations. We described the process of providing precise genetic counseling according to the fetus’ pathogenic evidence, leading to a decision whether to terminate the abnormal gestation, and informed those three families of professional fertility counseling and guidance for the next gestation.

## Data Availability

The original contributions presented in the study are publicly available. This data can be found here: https://doi.org/10.6084/m9.figshare.24598266.v1.
