# Supplementary material for: Identification of two novel and one rare mutation in DYRK1A and prenatal diagnoses in three Chinese families with intellectual Disability-7
Source: Front Genet. 2023 Dec 20;14:1290949. doi: 10.3389/fgene.2023.1290949 (PMC10765505; doi:10.3389/fgene.2023.1290949)
Supplement: Supplementary file 1 [file Table1.docx]

| **Supplementary TABLE 1 \|** Incidental findings of insufficient pathogenic evidence genes detected for the three probands in this study by trio-WES | | | | | | | |
| --- | --- | --- | --- | --- | --- | --- | --- |
|  | Gene | Chromosome /exon location | Transcript number/  nucleotide variantion/  amnio acid change | Pathogenic classification | Zygotic state | Inherited  pattern | Related disease |
|  |  |  |  |  |  |  |  |
| Proband of c.1159C>T | *ADCY1* | Chr7/  Exon9 | NM_021116/  c.1789C>T/  p.R597W | LP | Het | AR | Autosomal recessive hereditary hearing loss Type 44 |
|  | *ASPM* | Chr1/  Exon16 | NM_018136/  c.3812G>A/  p.R1271Q | LP | Het | AR | Autosomal recessive hereditary microcephaly Type 5 |
|  | *CASC5* | Chr15/  Exon21 | NM_170589/  c.6326C>A/  p.T2109N | VUS | Het | AR | Autosomal recessive hereditary microcephaly Type 4 |
|  | *IGF1* | Chr12/  Exon12 | NM_001111283/  c.*4463C>A/  - | VUS | Hom | AR | Growth retardation with hearing loss and intellectual disability casused by IGF1 deficiency |
|  | *LOXHD1* | Chr18/Exon32 | NM_144612/  c.4955T>C/  p.I1652T | LP | Het | AR | Autosomal recessive hereditary hearing loss Type 77 |
|  | *MOCS2* | Chr5/Exon5 | NM_004531/  c.229A>G/  p.T77A | LP | Het | AR | Deficiency of Complementary cofactor Group B |
|  | *PAFAHIB1* | Chr17/Exon2 | NM_000430/  c.-157G>A/  - | VUS | Het | - | Anencephalic malformation type 1/ Ectopic subcortical banded gray matter |
|  | *PNPT1* | Chr2/Exon28 | NM_033109/  c.*152delA/  - | VUS | Het | AR | Autosomal recessive hereditary hearing loss Type 70 |
|  |  |  | NM_033109/  c.*229_*232delAAGT/  - |  | Hom |  |  |
|  |  |  | NM_033109/  c.*258_*261delCATA/  - |  | Het |  |  |
|  | *SMPD1* | Chr11/Exon2 | NM_000543/  c.899C>T/  p.T300I | LP | Het | AR | Niemann-Pick Disease Type A |
|  |  | Chr11/Exon4 | NM_000543/  c.1307G>A/  p.S436N |  |  |  |  |
|  | *TUBB3* | Chr16/Exon1 | NM_006086/  c.-115C>G/  - | LP | Het | AD | Compound cortical dysplasia with other brain developmental abnormalities Type 1 |
|  |  | Chr16/Exon4 | NM_001197181/  c.*286A>T/  - |  |  |  |  |
|  |  |  |  |  |  |  |  |
| Proband of c.1309C>T | *NALCN* | Chr13/Exon3 | NM_052867.2/  c.194C>T/  p.P65L | VUS | Het | AD | Congenital limb facial contracture, hypotonia and developmental delay |
|  |  |  |  |  |  | AR | Infantile hypotonia with impaired psychomotor development and special facial features Type 1 |
|  | *NALCN* | Chr13/Exon8 | NM_052867.2/  c.874A>G/  p.S292G | VUS | Het | AD | Congenital limb facial contracture, hypotonia and developmental delay |
|  |  |  |  |  |  | AR | Infantile hypotonia with impaired psychomotor development and special facial features Type 1 |
|  |  |  |  |  |  |  |  |
| Proband of exon3_exon4del | *ARX* | ChrX/- | NM-139058.2/  c.592G>A/  p.V198I | VUS | Het | XL | 1.X-linked anencephalic malformation Type 2 |
|  |  |  |  |  |  |  | 2.Brain edema with genital abnormalities |
|  |  |  |  |  |  |  | 3.Proud syndrome |
|  |  |  |  |  |  |  | 4.Partington syndrome |
|  |  |  |  |  |  |  | 5.Early infantile epileptic encephalopahty |
|  |  |  |  |  |  |  | 6.X linked mental retardation |
|  | *1.C16orf52*  *2.CDR2*  *3.EEF2K*  *4.POLR3E*  *5.VWA3A* | Chr16:g.21812007_22547861  (GRCh37/hg19)/- | - | Pathogenic | Het | - | 1.Developmental delay  2.Mental retardation 3.Language backwardness  4.Facial abnormalities  5.Microcephaly  6.Congenital heart defects  7.Schizophrenia |
|  | *UQCRC2* | Chr16:g.21812007_22547861  (GRCh37/hg19)/- | - | Pathogenic | Het | - | 1.Mitochondrial encephalopathy  2.Psychomotor delay  3.Ataxia  4.Liver dysfunction  5.Metabolic acidosis  6.Renal tubular lesions  7.Muscle weakness  8.Exercise intolerance |
| Chr: chromosome, Het: heterozygote, Hom: homozygote, LP: likely-pathogenic, VUS: variant of uncertain significance. | | | | | | | |
